# A novel modeling approach for a generalizable photo-Fenton-based degradation of organic compounds

**DOI:** 10.1007/s11356-020-08616-4

**Published:** 2020-04-23

**Authors:** Francesca Audino, Montserrat Pérez-Moya, Moisès Graells, Antonio Espuña, Bela Csukas, Monika Varga

**Affiliations:** 1 Chemical Engineering Department, Escola d’Enginyeria de Barcelona Est (EEBE), Universitat Politècnica de Catalunya, Av. Eduard Maristany, 16, 08019 Barcelona, Spain; 2 Research Group on Process Network Engineering, Institute of Methodology, Kaposvar University, 40 Guba S, Kaposvar, 7400 Hungary

**Keywords:** AOP, Generalizable remediation mechanism, Multiple target compounds, Contaminants of emerging concern, Programmable process structure

## Abstract

This work aims at proposing and validating a model that can be exploited for the future development of industrial applications (e.g., process design and control) of Fenton and photo-Fenton processes. Hence, a compromise modeling solution has been developed between the non-generalizable accuracy of the first principles models (FPMs) and the oversimplification of the empirical models (EMs). The work presents a novel model of moderate complexity that is simplified enough to be generalizable and computationally affordable, while retaining physical meaning. The methodology is based on a general degradation mechanism that can be algorithmically generated from the carbon number of the target compound, as well as from the knowledge of two kinetic parameters, one for the faster initial rate and the other one for the subsequent degradation steps. The contaminant degradation mechanism has been combined with an appropriately simplified implementation of the well-known Fenton and photo-Fenton kinetics. This model describes the degradation not only of the target compound and of the oxidant, but also of total organic carbon (TOC), which is used to define the overall quality of the water. Experimental design techniques were used along with a non-conventional modeling methodology of programmable process structures (PPS). This novel modeling approach was applied and validated on the degradation of three model compounds. A successful prediction of the evolution of the contaminants H_2_O_2_ and TOC was confirmed and assessed by the root mean square error (RMSE).

## Introduction

A notable experimental effort has been spent in the last 20 years in investigating the advanced oxidation processes (AOPs), being an effective alternative to conventional biological processes for the treatment of toxic and recalcitrant wastewaters, and for the removal of contaminants of emerging concern (CECs) (Miklos et al. 2018).

A recent review by Mazivila et al. (2019) presents an interesting overview on AOPs, dividing them into a classical approach with emphasis on Fenton, photo-Fenton, and ozonation processes, all based on the generation of highly reactive hydroxyl radicals, and a novel approach. Among the new perspectives of AOPs, there are the ones based on the generation of emerging reactive sulfate (SO_4_^·−^) radicals, advanced electrochemical oxidation technologies (such as electro-Fenton and electro-photo-Fenton), nanocatalytic heterogeneous Fenton technology, and semiconductor photocatalysis (TiO_2_/UV), as well as the combination of processes involving at least one AOP.

In particular, among the classical AOPs, the photo-Fenton process has attracted special attention of the scientific community with an actual number of scientific papers related to photo-Fenton process of about 2700, as recently highlighted by Cabrera Reina et al. (2020). Scientific interest on photo-Fenton is due to the possibility of developing cost-effective treatment systems by exploiting solar energy and because it has proved to be one of the most promising AOPs in the treatment of industrial wastewaters. As a matter of fact, a review by Rahim Pouran et al. (2015) shows that most of the photo-Fenton applications that can be found in literature refer to the treatment of high-strength organic wastewaters. However, photo-Fenton has also showed good performances in the removal of micropollutants and CECs (Miralles-Cuevas et al. 2014; Villegas-Guzman et al. 2017).

Nevertheless, the transition from a pure experimental approach to a model-based approach is mandatory to allow process design, monitoring, control, and eventually process automation on which industrial applications are generally based on.

The literature review reveals that the proposed models can be enclosed in two main categories: first principles models (FPMs) and empirical models (EMs).

Some FPMs rely on the detailed kinetic modeling of Fenton and photo-Fenton processes and follow the scheme proposed by Kang et al. (2002) based on three main groups of reactions (an inorganic chemistry core; interactions between target compounds, intermediates, and Fenton reactants; and finally the interactions between iron species and the intermediates). The work by Simunovic et al. (2011) is a clear example of this class of FPMs. On the other hand, Alfano et al. (1986) implemented a modeling approach based on rigorous radiation models to compute the spatial distribution of the absorbed photons inside the reactor and on simplified kinetic reaction schemes. Following this line, a recent work by Audino et al. (2019) has proposed a kinetic study describing Fenton and photo-Fenton degradation of paracetamol in an annular photo-reactor, including the local volumetric rate of photon absorption effect. The work by Soriano-Molina et al. (2018) proposed a mechanistic model of solar photo-Fenton.

The FPMs are accurate models that aim at a full understanding of the chemical process. However, two main weak points arise, limiting their applicability in the real world. On one hand, the resulting computational effort might be unaffordable. On the other hand, the high complexity of Fenton and photo-Fenton systems, involving many reaction mechanisms that are still under investigation, makes it difficult to develop a comprehensive FPM that can include all the involved reaction mechanisms for more general cases of various and multiple targets.

Regarding the empirical models, in literature, it is possible to find preliminary works proposing the use of response surface platform for regression models (Pérez-Moya et al. 2008). More recently, semi-empirical models combining a first principles modeling approach with empirical observations have been proposed by Cabrera Reina et al. (2012, 2015). Finally, advanced multivariate techniques named data-based modeling (DBM) can also be found (Shokry et al. 2015, 2017).

Empirical models present some disadvantages that mainly rely on the oversimplification of the complex nonlinear behavior of the Fenton processes, as well as the limited capabilities of correlation of large sets of process variables. In this regard, the advanced techniques on which DBM is based on can allow a better capture of the nonlinear system behaviors; however, in this case, the physical understanding of the chemical process is not allowed.

Moreover, to the best of our knowledge, all these models have been proposed, trained, and validated for a specific target compound. The high complexity of the Fenton and photo-Fenton system makes it difficult to establish a coupling with the decontamination of quite different organic compounds. Hence, the modeling effort should face the need of implementing general enough models to allow describing the photo-Fenton degradation of any wastewater, containing single or multiple organic compounds and that can take into account their integrated action.

In the present work, starting from well-known FPMs, a set of hypotheses, based on empirical observations of the Fenton and photo-Fenton system behaviors, have been proposed with the aim of obtaining a model of medium complexity that can be simplified enough to be computationally affordable but without losing its physical meaning. Another challenge that we faced was modeling TOC evolution, a lumped parameter that is used to define the overall quality of the water.

The proposed novel modeling approach has been validated for the degradation of three target compounds. Paracetamol (PCT, C_8_H_9_NO_2_) is the first selected target compound, being the most widely used antipyretic and analgesic. As the second model compound, sulfaquinoxaline sodium salt (SQX, C_14_H_11_N_4_NaO_2_S) is selected because it is a potential food contaminant in animal products. Finally, formic acid (FA, CH_2_O_2_) is studied as the third target compound since it is a by-product of the degradation of many hazardous organic compounds and belongs to the group of carboxylic acids that are hardly degradable even by chemical oxidation (Salazar et al. 2017).

In this work, the design of experiments (DOE) technique was used to select the proper set of Fenton and photo-Fenton experiments to be performed in order to properly describe the system behavior for the different selected targets. The DOE was then combined with a non-conventional methodology named direct computer mapping (DCM) (Csukás et al. 1999, 2013) based programmable process structures (PPS) (Varga et al. 2016; Varga and Csukas 2017; Varga et al. 2017) that was used for modeling and simulation-based analysis of the experimental system.

## Experimental

### Reagents and chemicals

Paracetamol and sulfaquinoxaline sodium salt of 98% and 95% purity, respectively, purchased from Sigma-Aldrich (St. Louis, MO, USA), and formic acid (98%) purchased from Honeywell Fluka (Morris Plains, NJ, USA) were used as target compounds. Reagent-grade hydrogen peroxide (H_2_O_2_) (33% w/v) from Panreac Química SLU (Barcelona, Spain) and iron sulfate (FeSO_4_·7H_2_O) from Merck (Kenilworth, NJ, USA), adopted as the ferrous ion (Fe^2+^) source, were used as Fenton reagents. HPLC gradient-grade methanol (MeOH) purchased from J.T. Baker Inc. (Phillipsburg, NJ, USA) and filtered milliQ-grade water were used as HPLC mobile phases. High-purity (> 99%) ascorbic acid from Riedel de Haën (Seelze, Germany), 0.2% 1,10-phenanthroline from Scharlab SL (Barcelona, Spain), sodium acetate anhydrous, and 95–98% sulfuric acid, both from Panreac Química SLU (Barcelona, Spain), were used to perform measurements of iron species. Hydrogen chloride HCl 37% from J.T. Baker Inc. (Phillipsburg, NJ, USA) was used to adjust the initial pH. Deionized water with a conductivity lower than 1.25 μs was provided by Adesco S.A. (Barcelona, Spain) and was used as water matrix in all experiments.

### Analytical determinations

Measurements of total organic carbon (TOC) concentrations were performed with a Shimadzu VCHS/CSN TOC analyzer (Shimadzu; Kyoto, Japan) and samples were taken every 15.0 min (the measuring time of the equipment being 15.0 min). The samples were maintained refrigerated after extraction by submerging the flask in ice during the whole sampling time, with the aim of slowing down the degradation of the organic matter since the cooling of the samples to 2–5 °C is a preservation technique in case of measuring organic content (Janusz Pawliszyn 2002).

Target compound concentrations were measured with an HPLC Agilent 1200 series with UV-DAD array detector (Agilent Technologies, Santa Clara, CA, USA). In particular, paracetamol concentration ([PCT]) was determined using an Akady 5 μm C-18150 × 4.6 mm column, maintained at 25.0 °C as stationary phase, and a mixture of methanol:water (25:75) flowing at 0.4 mL min^−1^ as mobile phase. The diode array detector was set at 243 nm. Under these conditions, retention time was 9.0 min. Samples were taken at 0.0, 1.5, 2.5, 5.0, 7.5, 10.0, and 15.0 min and were previously treated with methanol (in proportion 50:50) in order to stop further degradation of PCT, being a well-known HO^•^ scavenger (Tadolini and Cabrini 1988; Múčka et al. 2013; Minella et al. 2018).

For the determination of SQX concentration ([SQX]), the same Akady 5 μm C-18150 × 4.6 mm column, kept at 25.0 °C, was used as stationary phase, while methanol and 0.1% (v/v) formic acid (55:45, v/v) were used as the mobile phase flowing at 1.5 mL min^−1^. In this case, the diode array detector was set at 250 nm. Under these conditions, retention time was 2.0 min. In this case, samples were taken at 0.0, 2.0, 5.0, 10.0, 15.0, 20.0, 25.0, 30.0, 45.0, 60.0, 75.0, 90.0, and 120.0 min, and were previously treated with methanol (in proportion 50:50) in order to stop the oxidation reactions.

In the case of formic acid (FA), only the TOC concentration was followed during the treatment span.

Finally, measurements of H_2_O_2_ and iron species concentrations were performed with a Hitachi U-2001 UV-VIS spectrophotometer (Hitachi, Tokyo, Japan). Hydrogen peroxide concentration ([H_2_O_2_]) was determined following the spectrophotometric technique described by Nogueira et al. (2005). This technique is based on the measurement of the absorption at 450 nm of the complex formed after reaction of H_2_O_2_ with ammonium metavanadate. In this case, samples were taken every 5.0 min until a reaction time of 30.0 min and then every 15.0 min until the end of the assay.

The iron species (Fe^2+^, Fe^3+^, Fe^TOT^) were analyzed using the 1,10-phenantranoline method following ISO 6332:^1988^, based on the absorbance measurements of the Fe^2+^-phenantroline complex at 510 nm. To measure total iron concentration, ascorbic acid must be used so as to convert all the ferric ions (Fe^3+^) to ferrous ions (Fe^2+^). Then, through the difference, ferric ion concentration could be determined. In this case, samples were taken every 5.0 min until a reaction time of 30.0 min, and then every 15.0 min until the end of the assay.

### Pilot plant and experimental protocol

The pilot plant that was used to perform Fenton and photo-Fenton assays is a 15.0-L system composed of an annular photo-reactor with an irradiated volume (*V*_IRR_) of 1.5 L and connected to a glass jacketed reservoir tank, both provided by Thermo Fisher Scientific, Barcelona, Spain.

The annular photo-reactor is equipped with an Actinic BL TL-DK 36 W/10 1SL lamp (UVA-UVB) provided by Barcelona LED (Barcelona, Spain), and the incident photon flux, measured by Yamal-Turbay et al. (2015) using potassium ferrioxalate actinometry (Murov et al. 1993), resulting in *E* = 3.36 × 10^−4^ Einstein min^−1^ (300 and 420 nm). The pilot plant is also equipped with sensors (conductivity, dissolved oxygen, oxidation reduction potential, and pH probes) connected to a programmable logic controller (PLC) that allows data acquisition and process control and to a SCADA system that allows data management.

Finally, a flowmeter is available for the control of the recirculation flow rate.

**Fig. 1:**
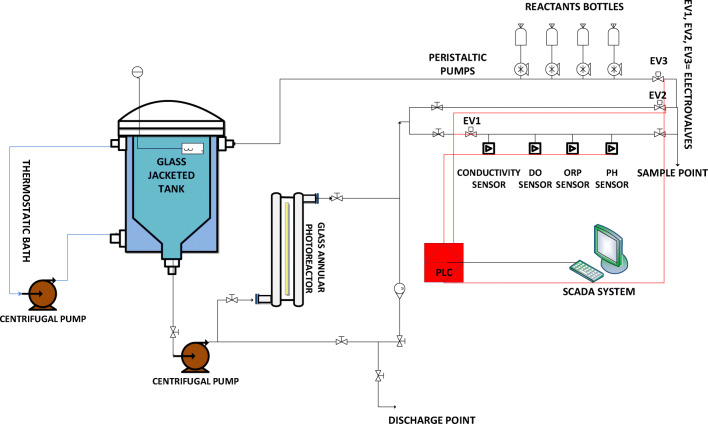
Schematic view of the pilot plant

A schematic view of the pilot plant is shown in Fig. 1.

Regarding the experimental protocol, the glass reservoir was first filled with 10.0 L of deionized water, and then after 10.0 min of recirculation, 4.9 L of deionized water in which the model contaminant was previously dissolved was added and recirculated for 15.0 min with the aim of ensuring a good homogenization of the matrix. After that, a sample was taken to measure the initial concentrations of TOC and the target compound. Then, after the pH was adjusted to 2.8 ± 0.2, the remaining 0.1 L of deionized water, in which iron sulfate was previously dissolved, was filled. After 10.0 min more of recirculation, another sample was taken with the aim of checking the initial concentration of the iron species ([Fe^2+^]^0^, [Fe^3+^]^0^, [Fe^TOT^]^0^). Finally, in the case of performing photo-Fenton experiments, the light was switched on 10.0 min before starting the experiment in order to allow the lamp to stabilize. The final step is the addition of hydrogen peroxide that makes the experiments start and that was added manually and all the once.

### Experimental design

As the first instance, a set of blank assays without any target compound (BLANK 1–4 in Table 1) was carried out in order to help the estimation of the kinetic parameters referring to the Fenton and photo-Fenton mechanisms, as well as the inefficient elimination of free radicals, in the proposed simplified first principles model. In particular, in this case, the initial concentration of ferrous ion [Fe^2+^]^0^ was set to 10.0 mg L^−1^ (corresponding to 0.18 mmol L^−1^), or rather to the maximum legal value in wastewaters in Spain (DOGC 2003). The initial concentration of hydrogen peroxide ([H_2_O_2_]^0^) was set to 378.0 mg L^−1^, corresponding to 11.12 mmol L^−1^. The latter was chosen being the highest value of the oxidant concentration among the ones used to perform the experiments with the target compounds that will be detailed in the following sections.

**Table 1:** Set of blank assays performed without any target compound

ID	[H_2_O_2_]^0^ (mmol L^−1^)	[Fe^2+^]^0^ (mmol L^−1^)	pH	*T* (°C)	*λ* (nm)	*V*_IRR_ (L)
BLANK_1	11.12	0.00	2.8 ± 0.2	28.0 ± 2.0	300–420	0.0
BLANK_2	11.12	0.00	2.8 ± 0.2	28.0 ± 2.0	300–420	1.5
BLANK_3	11.12	0.18	2.8 ± 0.2	28.0 ± 2.0	300–420	0.0
BLANK_4	11.12	0.18	2.8 ± 0.2	28.0 ± 2.0	300–420	1.5

Then, a set of experiments using paracetamol (PCT, C_8_H_9_NO_2_) as the target compound was carried out. A subset of these experiments was used to fit the proposed model to the specific degradation mechanism. Another subset of the experiments in Table 2 was used for the model validation.

**Table 2:** Design of experiments comprising the assays using PCT (C_8_H_9_NO_2_) as the target compound. Performed for an initial PCT concentration of [PCT]^0^ = 0.26 mmol L^−1^ corresponding to an initial TOC concentration of [TOC]^0^ = 25.92 mg L^−1^

ID	[H_2_O_2_]^0^ (mmol L^−1^)	[Fe^2+^]^0^ (mmol L^−1^)	pH	*T* (°C)	*λ* (nm)	*V*_IRR_ (L)
BLANK_5	11.12	0.00	2.8 ± 0.2	28.0 ± 2.0	300–420	0.0
BLANK_6	0.00	0.18	2.8 ± 0.2	28.0 ± 2.0	300–420	0.0
BLANK_7	11.12	0.00	2.8 ± 0.2	28.0 ± 2.0	300–420	1.5
BLANK_8	0.00	0.18	2.8 ± 0.2	28.0 ± 2.0	300–420	1.5
BLANK_9	0.00	0.00	2.8 ± 0.2	28.0 ± 2.0	300–420	1.5
EXP1_PCT	2.78	0.09	2.8 ± 0.2	28.0 ± 2.0	300–420	0.0
EXP2_PCT	2.78	0.13	2.8 ± 0.2	28.0 ± 2.0	300–420	0.0
EXP3_PCT	2.78	0.18	2.8 ± 0.2	28.0 ± 2.0	300–420	0.0
EXP4_PCT	5.56	0.09	2.8 ± 0.2	28.0 ± 2.0	300–420	0.0
EXP5_PCT	5.56	0.13	2.8 ± 0.2	28.0 ± 2.0	300–420	0.0
EXP6_PCT	5.56	0.18	2.8 ± 0.2	28.0 ± 2.0	300–420	0.0
EXP7_PCT	11.12	0.09	2.8 ± 0.2	28.0 ± 2.0	300–420	0.0
EXP8_PCT	11.12	0.13	2.8 ± 0.2	28.0 ± 2.0	300–420	0.0
EXP9_PCT	11.12	0.18	2.8 ± 0.2	28.0 ± 2.0	300–420	0.0
EXP10_PCT	2.78	0.09	2.8 ± 0.2	28.0 ± 2.0	300–420	1.5
EXP11_PCT	2.78	0.13	2.8 ± 0.2	28.0 ± 2.0	300–420	1.5
EXP12_PCT	2.78	0.18	2.8 ± 0.2	28.0 ± 2.0	300–420	1.5
EXP13_PCT	5.56	0.09	2.8 ± 0.2	28.0 ± 2.0	300–420	1.5
EXP14_PCT	5.56	0.13	2.8 ± 0.2	28.0 ± 2.0	300–420	1.5
EXP15_PCT	5.56	0.18	2.8 ± 0.2	28.0 ± 2.0	300–420	1.5
EXP16_PCT	11.12	0.09	2.8 ± 0.2	28.0 ± 2.0	300–420	1.5
EXP17_PCT	11.12	0.13	2.8 ± 0.2	28.0 ± 2.0	300–420	1.5
EXP18_PCT	11.12	0.18	2.8 ± 0.2	28.0 ± 2.0	300–420	1.5

In particular, Fenton (dark conditions, *V*_IRR_ = 0.0 L) and photo-Fenton (irradiated conditions, *V*_IRR_ = 1.5 L) experiments were performed fixing the initial concentration of PCT ([PCT]^0^ = 40.00 mg L^−1^) corresponding to 0.26 mmol L^−1^ and to an initial TOC concentration ([TOC]^0^ = 25.92 mg L^−1^), and changing the initial concentrations of ferrous ion [Fe^2+^]^0^ and hydrogen peroxide [H_2_O_2_]^0^.

The value of the initial PCT concentration is higher than that found in wastewaters and surface waters (Ternes, 1998; Kolpin et al. 2002; Lapworth et al. 2012), but it allows simulating the treatment of real paracetamol wastewater characterized by higher PCT, TOC, and chemical oxygen demand (COD) concentrations (Cabrera Reina et al. 2012; Dalgic et al. 2016).

The stoichiometric value of hydrogen peroxide concentration to achieve total mineralization of 0.26 mmol L^−1^ of PCT, when H_2_O_2_ is considered to be the only oxidant in the media, is 5.56 mmol L^−1^ (Eq. (1)):

1 $$ {\mathrm{C}}_8{\mathrm{H}}_9{\mathrm{NO}}_2+21{\mathrm{H}}_2{\mathrm{O}}_2\to 8{\mathrm{C}\mathrm{O}}_2+25{\mathrm{H}}_2\mathrm{O}+{\mathrm{H}}^{+}+{\mathrm{NO}}_3^{-} $$

In this case, a 2^2^ factorial design was applied in order to define the set of experiments to be performed. The two selected factorials were the Fenton reagents (Fe^2+^ and H_2_O_2_).

The initial ferrous ion concentration was varied between 0.18 mmol L^−1^ as the maximum value of the factorial design (+ 1), and half such value, 0.09 mmol L^−1^, as the minimum value of the factorial design (− 1), and one and a half times less, 0.13 mmol L^−1^, as the central value of the factorial design (0).

The initial hydrogen peroxide ion concentration was varied between half and twice (2.78–11.12 mmol L^−1^) the stoichiometric dose as maximum and minimum values of the factorial design (− 1, + 1). The stoichiometric dose (5.56 mmol L^−1^) corresponds to the central value of the factorial design (0).

The resulting design of experiment in the case of PCT is presented in Table 2 in which are reported all the specific conditions (pH, *T*, *λ*, and *V*_IRR_) of the experiments that were performed. Especially, the experiments from EXP1_PCT to EXP9_PCT are the experiments performed under dark conditions while those from EXP10_PCT to EXP_18_PCT are the ones performed under irradiated conditions.

Moreover, a set of preliminary standard blank assays, but with the presence of the target compound, was also performed and it is presented in Table 2 as well. The aim of these standard blank assays was to check the role of the oxidant (BLANK 5 and BLANK 7 experiments in Table 2) and the role of the catalyst (BLANK 6 and BLANK 8 experiments in Table 2) under both dark and irradiated conditions and the possible direct photolysis of PCT (BLANK 9 experiment in Table 2).

Next, a new target compound, the sulfaquinoxaline sodium salt (SQX, C14H11N4NaO2S), was used for validating the novel modeling approach that was proposed for PCT.

In this case, the aim was proving that the proposed methodological approach allows obtaining a generalizable solution that can be used to describe the photo-Fenton degradation of any kind of target compound.

Hence, in this case, to estimate the kinetic parameters of the relative degradation mechanism, a set of six experiments, three Fenton (dark conditions) and three photo-Fenton (irradiated conditions), was performed.

The initial concentration of SQX was fixed to 25.00 mg L^−1^ corresponding to 0.08 mmol L^−1^ and to an initial concentration of TOC ([TOC]^0^ = 13.72 mg L^−1^). The initial concentration of ferrous ion was also fixed and set to 0.18 mmol L^−1^. Conversely, the initial concentration of hydrogen peroxide was varied.

Again, a 2^2^ factorial design was applied. The initial concentration of hydrogen peroxide was varied between the stoichiometric value (3.47 mmol L^−1^ as the minimum factorial value, − 1) to achieve total mineralization of 0.08 mmol L^−1^ of SQX (according to the stoichiometric reaction shown in Eq. (2)) and one and a half times (5.24 mmol L^−1^, as the central factorial value, 0) and twice (6.94 mmol L^−1^ as the maximum factorial value, + 1) such value.

2 $$ {\mathrm{C}}_{14}{\mathrm{H}}_{11}{\mathrm{N}}_4{\mathrm{N}\mathrm{aO}}_2\mathrm{S}+45{\mathrm{H}}_2{\mathrm{O}}_2\to 14{\mathrm{C}\mathrm{O}}_2+47{\mathrm{H}}_2\mathrm{O}+4{\mathrm{H}\mathrm{NO}}_3+\mathrm{NaOH}+{\mathrm{H}}_2{\mathrm{SO}}_4 $$

The resulting design of experiment in the case of SQX is shown in Table 3, in which all the specific conditions (pH, *T*, *λ*, and *V*_IRR_) of the experiments that were performed, is reported. Especially, the experiments from EXP1_SQX to EXP3_SQX are the experiments performed under dark conditions while those from EXP4_SQX to EXP6_SQX are the ones performed under irradiated conditions.

**Table 3:** Design of experiments comprising the assays using SQX (C_14_H_11_N_4_O_2_S) as the target compound. Performed for an initial SQX concentration of [SQX]^0^ = 0.08 mmol L^−1^ corresponding to an initial TOC concentration of [TOC]^0^ = 13.72 mg L^−1^

ID	[H_2_O_2_]^0^ (mmol L^−1^)	[Fe^2+^]^0^ (mmol L^−1^)	pH	*T* (°C)	*λ* (nm)	*V*_IRR_ (L)
BLANK_10	6.94	0.00	2.8 ± 0.2	28.0 ± 2.0	300–420	0.0
BLANK_11	0.00	0.18	2.8 ± 0.2	28.0 ± 2.0	300–420	0.0
BLANK_12	6.94	0.00	2.8 ± 0.2	28.0 ± 2.0	300–420	1.5
BLANK_13	0.00	0.18	2.8 ± 0.2	28.0 ± 2.0	300–420	1.5
BLANK_14	0.00	0.00	2.8 ± 0.2	28.0 ± 2.0	300–420	1.5
EXP1_SQX	3.47	0.18	2.8 ± 0.2	28.0 ± 2.0	300–420	0.0
EXP2_SQX	5.24	0.18	2.8 ± 0.2	28.0 ± 2.0	300–420	0.0
EXP3_SQX	6.94	0.18	2.8 ± 0.2	28.0 ± 2.0	300–420	0.0
EXP4_SQX	3.47	0.18	2.8 ± 0.2	28.0 ± 2.0	300–420	1.5
EXP5_SQX	5.24	0.18	2.8 ± 0.2	28.0 ± 2.0	300–420	1.5
EXP6_SQX	6.94	0.18	2.8 ± 0.2	28.0 ± 2.0	300–420	1.5

In addition, as for PCT, a set of five preliminary standard blank assays was performed in order to check the possible direct photolysis of SQX (BLANK ASSAY 14 in Table 3). The role of Fenton reagents under both dark (BLANK ASSAY 10 and 11 in Table 3) and irradiated conditions (BLANK ASSAY 12 and 13 in Table 3) was investigated, as well.

Finally, the modeling approach was validated for the degradation of a third compound, formic acid (FA, CH_2_O_2_).

In this case, a set of only four experiments, two Fenton (dark conditions) and two photo-Fenton (irradiated conditions), was performed.

The initial concentrations of FA were fixed to 40.00 mg L^−1^ corresponding to 0.87 mmol L^−1^ and to an initial concentration of TOC, ([TOC]^0^ = 10.43 mg L^−1^). The initial concentration of ferrous ion was also fixed and set to 0.09 mmol L^−1^. Conversely, the initial concentration of hydrogen peroxide was changed between the stoichiometric value (0.88 mmol L^−1^) to achieve total mineralization of 0.87 mmol L^−1^ of FA (according to the stoichiometric reaction shown in Eq. (3)) and twice (1.76 mmol L^−1^) such value.

3 $$ \mathrm{C}{\mathrm{H}}_2{\mathrm{O}}_2+{\mathrm{H}}_2{\mathrm{O}}_2\to {\mathrm{CO}}_2+2{\mathrm{H}}_2\mathrm{O} $$

Additionally, another photo-Fenton experiment was performed by increasing the initial concentrations of ferrous ion and hydrogen peroxide to 0.18 mmol L^−1^ and 4.41 mmol L^−1^, respectively, so to test the effect of higher concentrations of the Fenton reagents.

The resulting design of experiment in the case of FA is shown in Table 4 in which are reported all the specific conditions (pH, *T*, *λ*, and *V*_IRR_) of the experiments that were performed. Especially, the experiments from EXP1_FA to EXP2_FA are the experiments performed under dark conditions while those from EXP3_FA to EXP5_FA are the ones performed under irradiated conditions.

**Table 4:** Design of experiments comprising the assays using FA (CH_2_O_2_) as the target compound. Performed for an initial FA concentration of [FA]^0^ = 0.87 mmol L^−1^ corresponding to an initial TOC concentration of [TOC]^0^ = 10.43 mg L^−1^

ID	[H_2_O_2_]^0^ (mmol L^−1^)	[Fe^2+^]^0^ (mmol L^−1^)	pH	*T* (°C)	*λ* (nm)	*V*_IRR_ (L)
EXP1_FA	0.88	0.09	2.8 ± 0.2	28.0 ± 2.0	300–420	0.0
EXP2_FA	1.76	0.09	2.8 ± 0.2	28.0 ± 2.0	300–420	0.0
EXP3_FA	0.88	0.09	2.8 ± 0.2	28.0 ± 2.0	300–420	1.5
EXP4_FA	1.76	0.09	2.8 ± 0.2	28.0 ± 2.0	300–420	1.5
EXP5_FA	4.41	0.18	2.8 ± 0.2	28.0 ± 2.0	300–420	1.5

All the experiments were performed for 120.0 min in batch mode with recirculation and ensuring perfect mixing conditions by setting the recirculation flow rate to 12.0 L min^−1^.

In particular, pH was continuously monitored in order to ensure that it was inside the range (2.5 < pH < 3.0–4.0) that, according to Pignatello (1992), Pignatello et al. (1999), and Pignatello et al. (2006), avoids iron precipitation and ensures the reduction of Fe^3+^ to Fe^2+^ (Fenton-like reaction) at an appreciable rate. Temperature was also continuously monitored and it was checked that it was inside the range *T* = 28.0 ± 2.0 °C.

## Model development

The modeling effort had to face two main challenges:

(i) To develop a model that can be exploited for future industrialization of Fenton and photo-Fenton processes. In particular, a model that can be simplified enough to be computationally affordable but without losing its physical meaning. Or, in other words, a compromise modeling solution between the unaffordable accuracy of the first principle models (FPMs) and the oversimplification of the empirical models (EMs).
(ii) To develop a rationally simplified but easily configurable and generally usable approach that can be applied for different wastewater systems containing single or multiple targets by also modeling a lumped parameter that can be used to define the overall quality of the water such as the TOC.

In the present work, starting from well-known FPMs, a set of hypotheses, rationally selected and based on empirical observations of the Fenton and photo-Fenton system behavior, have been proposed and are listed below:

### H1

The existence of a general target independent model of Fenton system, initiated by mixing H_2_O, H_2_O_2_, and Fe^2+^ and described by a set of kinetic reactions involving these compounds, as well as Fe^3+^, free radicals (at least HO^•^ and ${\mathrm{HO}}_2^{\bullet }$), and ions (at least H^+^ and OH^−^), was assumed as the core mechanism of the proposed model. The target invariant free radical production of Fenton process was described by the H_2_O_2_-driven Fe reaction cycle:

4 $$ {\mathrm{Fe}}^{3+}+{\mathrm{H}}_2{\mathrm{O}}_2\ \overset{{\mathrm{k}}_1}{\to }\ {\mathrm{Fe}}^{2+}+{\mathrm{H}}^{+}+{\mathrm{H}\mathrm{O}}_2^{\bullet } $$

5 $$ {\mathrm{Fe}}^{2+}+{\mathrm{H}}_2{\mathrm{O}}_2\overset{{\mathrm{k}}_2}{\to }\ {\mathrm{Fe}}^{3+}+{\mathrm{H}\mathrm{O}}^{-}+{\mathrm{H}\mathrm{O}}^{\bullet } $$

### H2

The core mechanism was assumed also to comprise the well-known surplus generation of HO^•^ in the lighted photo-Fenton system. The target invariant light initialized free radical production of the photo-Fenton process proposed by Cabrera Reina et al. (2012) was assumed:

6 $$ {\mathrm{Fe}}^{3+}+{\mathrm{H}}_2\mathrm{O}\ \overset{\mathrm{LIGHT},{\mathrm{k}}_0}{\to }\ {\mathrm{Fe}}^{2+}+{\mathrm{H}}^{+}+{\mathrm{H}\mathrm{O}}^{\bullet } $$

In this case, Cabrera Reina et al. (2012) proposed the following reaction scheme explicitly containing the UV irradiance (*I*):

7 $$ {r}_0={k}_0\ \left[{\mathrm{Fe}}^{3+}\right]\ I $$

and the kinetic parameter *k*_0_ of the photo-Fenton reaction was determined in line with data by Cabrera Reina et al. (2012).

### H3

In order to simplify the computational effort, the following fictitious first-order reaction model was heuristically assumed and validated to describe the inefficient elimination of the free radicals:

8 $$ {r}_{\mathrm{elimination}}:\kern1em \frac{\Delta \left[{\mathrm{HO}}^{\bullet}\right]}{\Delta \mathrm{t}}=-{k}_{\mathrm{elimination}}\ \left[{\mathrm{HO}}^{\bullet}\right] $$

Kinetic parameter (*k*_elimination_) is to be identified and validated.

In addition, there is an evident auxiliary equilibrium reaction:

9 $$ {\mathrm{H}}^{+}+{\mathrm{OH}}^{-}\ \overset{{\mathrm{K}}_{\mathrm{w}}}{\iff }{\mathrm{H}}_2\mathrm{O} $$

which was calculated according to the controlled pH and for a pH = 2.8, the change for [H^+^] resulted in 0.16 × 10^−2^ mol L^−1^.

### H4

As the first preliminary hypothesis, it was assumed that the degradation mechanism of the targeted organic compounds takes place only via the molecule breakage mechanism. It was supposed that the simplified general degradation mechanism of targeted organic compounds (*T*) and of their fragments (FR) may be interpreted as the series of consecutive breakage steps (*B*), comprising second-order binary reaction of the target and its fragments with free radicals. The number of these steps was assumed to be formally (and algorithmically) determined by the number of carbon atoms (NC) in the molecule to be decomposed. It was assumed that in the decomposition steps the targeted compounds and their fragments interact basically with free radical HO^•^ (main oxidant species) as also proposed by other authors, e.g., by Conte et al. (2012).

Based on such hypothesis, the following equations were derived:

10 $$ {\mathrm{FR}}_{\mathrm{b}-1}+{\mathrm{HO}}^{\bullet }\ \overset{{\mathrm{k}}_{\mathrm{b}-1}}{\to }\ 2\ {\mathrm{FR}}_{\mathrm{b}} $$

11 $$ {\mathrm{FR}}_{\mathrm{b}}+{\mathrm{HO}}^{\bullet }\ \overset{{\mathrm{k}}_{\mathrm{b}}}{\to }\ 2\ {\mathrm{FR}}_{\mathrm{b}+1} $$

Therefore, the reaction rate could be calculated as follows:

12 $$ \frac{\mathrm{d}\left[{\mathrm{FR}}_{\mathrm{b}}\right]}{\mathrm{d}\mathrm{t}}=2{\mathrm{r}}_{\mathrm{b}-1}-{\mathrm{r}}_{\mathrm{b}}=2\ \left[{\mathrm{FR}}_{\mathrm{b}-1}\right]\left[{\mathrm{HO}}^{\bullet}\right]{\mathrm{k}}_{\mathrm{b}-1}-\left[{\mathrm{FR}}_{\mathrm{b}}\right]\left[{\mathrm{HO}}^{\bullet}\right]{\mathrm{k}}_{\mathrm{b}} $$

with the initial conditions [FR_0_] = [*T*] and [FR_b_] = 0 while *b* = 1, 2,..., *B*, and *B* is the number of the consecutive breakage steps, which can be calculated from the number of carbon atoms NC according to constraints:

13 $$ B-1<{\log}_2\mathrm{NC}\ \leq\ B $$

### H5

As the first preliminary hypothesis, it was also supposed a faster initial rate of the degradation mechanism based on the hypothesis that the by-products are more hardly degradable than the parent compound. Hence, it was assumed that the breakage steps can be characterized by two target-specific parameters, as follows:

- *k*_target_ = *k*_0_ (*b* = 0) for the first breakage step of the targeted compound, and
- *k*_fragment_ = *k*_b_ (*b* = 1,2, ..., *B*) for all the following fragmentation steps.

The target-specific temperature-dependent parameters *k*_target_ and *k*_fragment_ have to be estimated and validated for the various targets, individually.

### H6

It was assumed that the concentration of the TOC can be calculated from the fictitious number of carbon atoms (FNC_b_) in the fragments originating from the parent compound (FR_b_). The number of fragments depends on the number of carbon atoms in the starting molecule, as follows:

14 $$ {FNC}_b= NC/{2}^b\to b=0,1,2,\dots, B\to {FNC}_0=T\left(t\arg et\right) $$

The approximate concentration of TOC can be calculated from these values as follows:

15 $$ \left[\mathrm{TOC}\right]={\sum}_{\mathrm{b}=0}^{\mathrm{B}}{\mathrm{FNC}}_{\mathrm{b}}{\mathrm{c}}_{\mathrm{b}} $$

Where *c*_b_ is the concentration of each of the produced fragments.

It must be noted that the target molecule and the modeled fragments produced from it contribute to the calculated TOC value.

### H7

In order to avoid infeasible stiffness that can be generated by the competitive reactions (i.e., of the different components) with HO^•^ proportional rates, normalized by the summarized rate of the reactions competing for radical HO^•^ were assumed:

16 $$ {r}_{\mathrm{i},\operatorname{mod}}=\frac{r_{\mathrm{i}}}{r_{\mathrm{elimination}}+\sum \limits_{\mathrm{b}=0}^{\mathrm{B}}{\mathrm{r}}_{\mathrm{b}}}\ast {\mathrm{r}}_{\mathrm{i}} $$

where the inefficient reaction of the free radicals HO^•^ (*r*_elimination_ according to H3) also participates in this competition.

Hence, the developed model presents two main steps: a core mechanism (Fenton, photo-Fenton, and inefficient reactions of the free radicals, defined by hypotheses H1, H2, and H3 and described by Eqs. (4)–(8), as well as a general contaminant degradation mechanism (usable for different target compounds, defined by hypotheses H4, H5, H6, and described by Eqs. (10)–(15)).

## Implementation of programmable process structures (PPS)

The non-conventional methodology of PPS (antecedent DCM version: Csukás et al. 1999; Csukás et al. 2013; recent PPS version: Varga et al. 2017; Varga and Csukas 2017) was applied for modeling and simulation-based analysis of the experimental system. The method has been already tried and validated for dynamic simulation-based analysis of multi-scale, hybrid processes in a broad range of applications from cellular signaling-based biosystems to agri-food and environmental process systems (Varga et al. 2016; Varga et al. 2017).

**Fig. 2:**
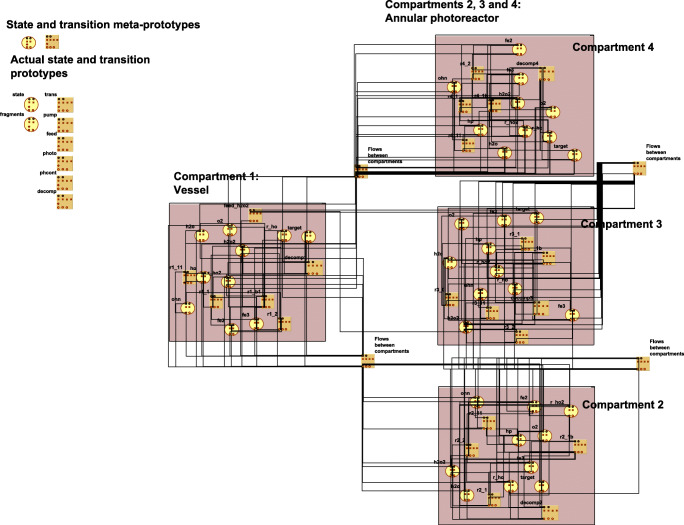
The generated structure (the expert interface) of the model

In line with the abovementioned former works, PPS can be generated from one state and one transition meta-prototype, as well as from the standardized description of the process network, automatically (see an example in Fig. 2). The meta-prototypes and the description of the process network, as well as the generated PPS, are prepared for the connection (message)-based communication between a state and the transition elements. State elements have dedicated output connectors for intensive properties and for signals, while transition elements have dedicated connectors to receive the data addressed to them. Similarly, transition elements have dedicated output connectors for the changes of extensive properties and for signals, while state elements have dedicated connectors to receive the data addressed to them.

The functionalities of the state and transition elements are declared by the locally executable program codes, embedded in the so-called case-specific prototype elements, derived as the copies of the general meta-prototypes. The model gets the initial values and the parameters of the generated, actual state and transition elements from an auxiliary database, following the structure of their case-specific prototypes, automatically.

**Fig. 3:**
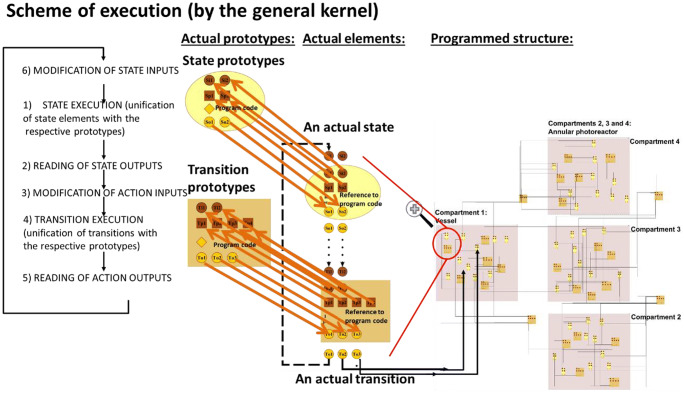
The execution cycle of PPS models by the general kernel

The execution (see an example in Fig. 3), as the connection-based communication among the state and transition elements of the programmed process structure, is solved by the general purpose kernel program.

Recent experimental implementation consists of:

- General kernel of model generator and simulator (platform-independent GNU-Prolog);
- GraphML-based model data files (can be edited with graph editors, e.g., with yEd Graph editor for GraphML);
- MS Excel user interface with VBA for initial data and parameters, as well as for visualization of simulated results.

In particular, in the present work, in order to follow the real hydro-dynamical conditions as well as to support possible future scaling up of the process, we compartmentalized the pilot plant into a circular set of four parts:

- Compartment [1] the tank reactor of 9.00 L, where the H_2_O_2_ reactant is fed and mixed with the previously mixed solution of the target compound and Fe^2+^;
- Compartment [2] of 2.25 L, connecting compartments [1] and [3];
- Compartment [3] of 1.50 L, representing the irradiated volume of the annular reactor;
- Compartment [4] of 2.25 L, connecting compartments [3] and [4].

Accordingly, it must be noted that:

- Compartment [1] is associated with the feeding of H_2_O_2_ that followed the fast but realistically finite time because it was observed that this short-term dosage better fits the experimented behavior of the system than starting from a calculated initial condition;
- The reaction in Eq. (6) was taken into account only in compartment [3] which is the only one associated with the irradiated volume of the pilot plant;
- All the other reactions were taken into account in the whole set of compartments ([1]–[4]).

In the actual model implementation, the following state and transition prototypes were defined:

- Two-state prototypes: one for the general Fenton components (core mechanism) and another one for the target-related components (prepared for the general contaminant degradation mechanism);
- Six transition prototypes: one for the Fenton system (Eqs. (4) and (5)), one for photo-Fenton reaction (Eq. (6)), one for pH-related dissociation equilibrium of water (Eq. (9)), one for target decomposition (Eqs. (10)–(15), one for H_2_O_2_ feeding, and one for recirculation of the water ensured by a recycle pumping between the compartments.

Figure 2 presents the PPS that was generated from the general state and transition meta-prototypes. In addition to these meta-prototypes, also the actually developed program prototypes, containing the local programs, are illustrated in Fig. 2 (upper left side).

All the abovementioned state elements and transition elements are summarized in Table 5.

**Table 5:** The state and transition elements of the generated structure

10 state elements	
Symbolic notations used in the model	Description
[fe2]	Fe^2+^
[fe3]	Fe^3+^
[hp]	H^+^
[ohn]	OH^−^
[h2o]	H_2_O
[h2o2]	H_2_O_2_
[r_ho]	HO^•^
[o2]	O_2_
Target	Actual used organic compound
f1, f2, f3, ...fB	Fragments
6 transition elements	
Symbolic notations used in the model	Description
trans	Fenton reactions (Eqs. (4), (5))
photo	Photo-Fenton reaction (Eq. (6))
phcont	pH-related dissociation equilibrium of water (Eq. (9))
decomp	Target decomposition (Eqs. (10)–(15))
feed_h2o2	Feeding of H_2_O_2_
pump1, pump2, pump3, pump4	Recycle pumping between the compartments

Figure 3 illustrates the execution scheme of the PPS models by the general kernel algorithm through the example of photo-Fenton model.

## Results and discussion

Once the model was formulated and the experimental designs were proposed for the selected model compounds, it was possible to proceed to the model training and validation step.

First, the estimation of the kinetic parameters of the core mechanism was carried out by using the data of the blank assays, performed without target compounds (Table 1).

Then, the model was trained and validated by using PCT as the target compound (assays are shown in Table 2). In particular, a subset of the assays in Table 2, or rather EXP1_PCT, EXP4_PCT, EXP5_PCT, EXP9_PCT, EXP10_PCT, EXP13_PCT, EXP14_PCT, and EXP18_PCT, was used for training while the remaining experiments were used for model validation.

Finally, the methodology was validated by estimating the kinetic parameters for the degradation of the other targets (SQX and FA) by using the respective experimental dataset (see Tables 3 and 4, respectively).

Considering model validation, it is to be noted that according to the model hypotheses H1, H2, and H3, the parameters of the target invariant free radical production of Fenton process were described by the H_2_O_2_-driven Fenton reaction cycle, as well as by the surplus generation of HO^•^ in the lighted photo-Fenton system. The respective model parameters from the limited amount of experiments without target were fixed as described in the section “Estimation of the kinetic parameters of the general core mechanism (Fenton, photo-Fenton, and inefficient elimination of the free radicals).”

Next, in the knowledge of the previously fixed general parameters, the identification and validation were limited to the 2 additional parameters, considering model hypotheses H4, H5, and H6, while 3 evaluation measures (NRMSE for target, TOC, and H_2_O_2_) were used.

For PCT (see the section “Estimation of the kinetic parameters of the degradation mechanism for the first target compound (PCT)”), more experiments were available to select identification (training) and validation experiments.

In the case of additional SQXNa and FA targets, fewer experiments were available, but they were supplied with some qualitative background knowledge from the PCT case. First, the “central” experiments were selected from the domain of the technological parameters, in order to find the appropriate parameter sets, while the trial and error search was controlled by the TOC > Target > H_2_O_2_ priority. The TOC > Target > H_2_O_2_ priority hypothesis was assumed due to the importance of describing the evolution of the TOC as a lumped parameter that gives an indication of the water quality in accordance with the main aim of developing a model that can be applied in different wastewater systems, containing single or multiple targets. Afterwards, the obtained solution was tested for the “terminal” experiments trying to find better parameter sets. Next, “central” cases were examined again and optionally parameters were refined. Finally, the whole set of experiments was simulated by fixing all the parameters that were estimated.

In the following sections, illustrative examples of the simulation results compared with experimental data are shown, including the case of the core mechanism (using the experimental data obtained performing the assays without target compound) and for each of the three selected targets. The comparison is evaluated by the RMSE, calculated by the following expression:

$$ {\mathrm{RMSE}}_i=\sqrt{\sum \limits_k{\left({\mathrm{y}}_{\mathrm{i}\mathrm{k}}-{\mathrm{y}}_{\mathrm{i}\mathrm{k}}^{\ast}\right)}^2/{\mathrm{n}}_{\mathrm{i}}} $$

root mean square error of the *i*th variable, where:

- *y*_ik_, value of the *k*th measurement of the *i*th variable
- $$ {y}_{\mathrm{ik}}^{\ast },\mathrm{value}\ \mathrm{of}\ \mathrm{the}\ {\mathrm{k}}^{\mathrm{th}}\ \mathrm{estimation}\ \mathrm{of}\ \mathrm{the}\ i\mathrm{th}\ \mathrm{variable}\ \left(\mathrm{model}\ \mathrm{prediction}\right) $$
- *i* = 1, 2, …I, the i^th^ element of the set of measured variables

The measured data, involved in the RMSE calculations, were the normalized concentrations of the target compound, TOC, and H_2_O_2_ (*I* = 3).

### Estimation of the kinetic parameters of the general core mechanism (Fenton, photo-Fenton, and inefficient elimination of the free radicals)

In this step, the kinetic parameters *k*_0_, *k*_1_, *k*_2_, and *k*_elimination_ of reactions in Eqs. (6), (4), (5), and (8), respectively, were determined by using the experimental data of the assays performed without target compounds (BLANK_1–4, Table 1).

As initialization values for *k*_0_, *k*_1_, and *k*_2_, the ones available in literature were adopted. Conversely, *k*_elimination_ was set as a free parameter to be estimated.

Especially:

- The initialization value for *k*_1_ was adopted from Walling and Goosen (1973) referring to a range *k*_1_ = 0.1–11.7 L mol^−1^ s^−1^;
- The initialization value for *k*_2_ was adopted from Simunovic et al. (2011) referring to a range *k*_2_ = 63.0–76.0 L mol^−1^ s^−1^;
- The initialization value for *k*_0_ was calculated from Cabrera Reina et al. (2012) using Eq. (7), considering I = 36 W m^−2^.

**Fig. 4:**
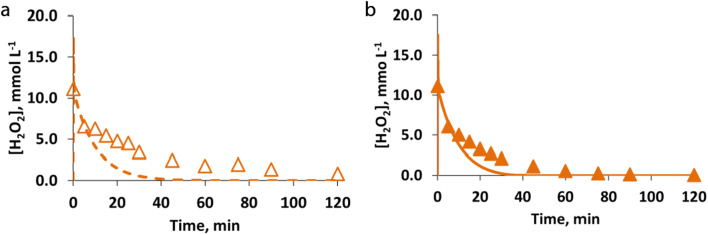
Evolution of measured (symbols) and calculated (line) concentrations of hydrogen peroxide in cases of blank experiments performed without the target compound ([Fe^2+^]^0^ = 0.18 mmol L^−1^, [H_2_O_2_]^0^ = 11.12 mmol L^−1^), under (**a**) dark (empty symbol and dashed line) conditions and (**b**) irradiated (solid symbol and solid line) conditions

The final estimated values are reported below:

The following Fig. 4 shows the comparison between measured and calculated results of hydrogen peroxide, obtained for the blank experiments without any target compound, named BLANK_3 and BLANK_4 (Table 1) referring to dark and irradiated conditions, respectively.

From the experimental results in Fig. 4, it can be observed that the use of radiation determines a small but characteristic increase of hydrogen peroxide consumption (e.g., after 10.0 min, in case of using radiation a 55% H_2_O_2_ consumption was observed, conversely a 44% consumption was experimented under dark conditions).

The hydrogen-peroxide consumption without the presence of contaminants is the consequence of cyclical reduction of Fe^3+^ and oxidation of Fe^2+^, both reacting with H_2_O_2_ (see Eqs. 4 and 5). However, radiation not only produces more useful HO^•^ radicals but also a higher reduction of Fe^3+^ to Fe^2+^ whose higher concentration determines an increase in H_2_O_2_ consumption.

The proposed core mechanism, with the estimated kinetic parameters, is able to describe this behavior even if it slightly overestimates the H_2_O_2_ consumption.

The RMSE value, calculated as the average value of the RMSE obtained for the dark conditions and the RMSE obtained for the irradiated conditions, resulting in 17%.

### Estimation of the kinetic parameters of the degradation mechanism for the first target compound (PCT)

In this step, two kinetic parameters needed to be estimated, namely:

- ${k}_{\mathrm{target}}^{\mathrm{PCT}}$ [L(mmol s)^−1^] for the first degradation step of the target compound;
- ${k}_{\mathrm{fragments}}^{\mathrm{PCT}}$ [L(mmol s)^−1^] for the following degradation steps of the fragments.

The kinetic parameters of the core mechanisms (*k*_0_, *k*_1_, *k*_2_, and *k*_elimination_) were assumed equal to those estimated in the previous step and presented in Table 6.

**Table 6:** Estimated values of the kinetic parameters of the core mechanism (Fenton, photo-Fenton, and inefficient elimination of the free radicals)

*k*_0_ (s^−1^)	*k*_1_ (L mol^−1^ s^−1^)	*k*_2_ (L mol^−1^ s^−1^)	*k*_elimination_ (s^−1^)
5.6 × 10^−2^	5.0	63.0	7.5 × 10^−2^

The parameter estimation was carried out using a subset of the experiments reported in Table 2, or rather: EXP1_PCT, EXP4_PCT, EXP5_PCT, EXP9_PCT, EXP10_PCT, EXP13_PCT, EXP14_PCT, and EXP18_PCT. The remaining experiments were used for model validation.

The specific PCT degradation mechanism and the estimated values of the relative kinetic parameters are summarized in Table 7.

**Table 7:** Degradation mechanism and estimated values of the related kinetic parameters for PCT

Target compound: PCT (C_8_H_9_NO_2_)	
Degradation mechanism	
Formal fragmentation	8 → 4 → 2 → 1
TOC value	TOC = 8∙c_1_ + 4∙c_2_ + 2∙c_3_
Number of carbon atoms, NC	NC = 8
Number of the consecutive breakage steps, B	*B* = 4
Target-specific model parameters	
${k}_{\mathrm{target}}^{\mathrm{PCT}}$ = 15.0 × 10^2^ L mol^-1^s^-1^ ${k}_{\mathrm{fragments}}^{\mathrm{PCT}}$ = 1.5 × 10^2^ L mol^-1^s^-1^	

As can be observed by the estimated values of the kinetic parameters in Table 7, the rate of the first degradation step resulted in one order magnitude higher than the rate of the subsequent degradation steps.

**Fig. 5:**
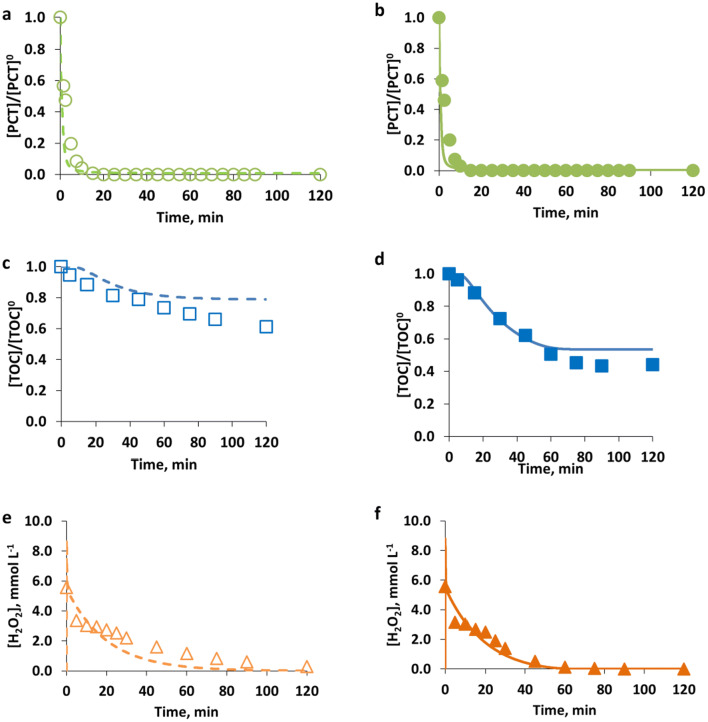
Model training results: comparison of measured (symbols) and simulated (lines) values of PCT (circle, **a** and **b**), TOC (square, **c** and **d**) presented as normalized values, and H_2_O_2_ (triangle, **e** and **f**) presented as concentration values (mmol L^−1^), obtained for [PCT]^0^ = 0.26 mmol L^−1^; [Fe^2+^]^0^ = 0.09 mmol L^−1^; [H_2_O_2_]^0^ = 5.56 mmol L^−1^ under dark (empty symbols and dashed lines, **a**, **c**, **e**) and irradiated (solid symbols and lines, **b**, **d**, **f)** conditions

An illustrative case of the model training for the case of EXP4_PCT and EXP_13_PCT (see Table 2) is shown in Fig. 5. Specifically, the comparison of the simulated and measured data for the different model components (PCT, TOC, H_2_O_2_) obtained in the case of using [PCT]^0^ = 0.26 mmol L^−1^; [Fe^2+^]^0^ = 0.09 mmol L^−1^; [H_2_O_2_]^0^ = 5.56 mmol L^−1^ under dark and irradiated conditions, respectively, is reported.

Model results in terms of PCT concentrations show a similar behavior under dark and irradiated conditions, as also shown by experimental results. In particular, modeling and experimental results showed a fast initial stepwise degradation of PCT that slows down later on by the decreased Fe^3+^ concentration and finally decays below the HPLC detection limit in about 15.0 min under both dark and irradiated conditions.

On the other hand, the effect of radiation was more evident by observing the TOC mineralization.

In particular, experimental results of the assay performed under irradiated conditions show that after 30.0 min a 28% TOC conversion was reached; conversely, under dark conditions, only 19% TOC conversion was attained.

The model is able to represent this behavior: under irradiated conditions and after 30.0 min of reaction, the model estimates a TOC conversion of 29%, while under dark conditions TOC conversion after 30.0 min resulted to be 12%.

Moreover, despite the greater differences observed during the first steps of the reaction, the radiation allowed attaining an enhancement of the final TOC conversion too. Under dark conditions, it was possible to attain a maximum TOC conversion of 39%, on the contrary; the use of radiation led to a final TOC conversion of 56%.

The model can describe also this behavior and in fact calculates a final TOC conversion of 22% in cases of dark conditions and of 47% in cases of irradiated conditions.

Finally, Fig. 5 f shows that similar results in terms of H_2_O_2_ consumption were observed under dark and irradiated conditions and this behavior was well described by the model. In this case, under dark conditions and after 10.0 min of reaction, the model estimates a 40% H_2_O_2_ consumption against a 46% real H_2_O_2_ consumption. Conversely, under irradiated conditions, the model estimates a 43% H_2_O_2_ consumption against a 45% real H_2_O_2_ consumption.

**Fig. 6:**
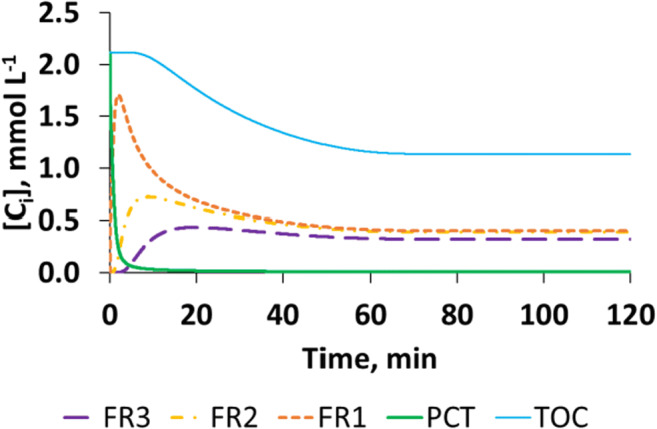
Simulated concentrations of the TOC, PCT, and the fragments originating from PCT (*C*_*i*_, *i* = PCT, TOC, FR1, FR2, FR3, expressed as mmol L^−1^) in the case of using [PCT]^0^ = 0.26 mmol L^−1^; [Fe^2+^]^0^ = 0.09 mmol L^−1^; [H_2_O_2_]^0^ = 5.56 mmol L^−1^ and irradiated conditions

Moreover, for the sake of completeness, an illustrative example of the fragments evolution is presented in Fig. 6. This shows the simulated values of the fragment concentrations, compared with the simulated evolution of PCT and TOC concentration in the case of EXP13_PCT or rather in the case of using [PCT]^0^ = 0.26 mmol L^−1^; [Fe^2+^]^0^ = 0.09 mmol L^−1^; [H_2_O_2_]^0^ = 5.56 mmol L^−1^ and irradiated conditions. We did not measure the intermediates, formed during the reactions; however, the sum of the PCT and fragment concentrations multiplied by the proper number of carbon atoms gives the simulated value of TOC, which is then compared with the experimental data of TOC concentrations.

Regarding the fragment evolution, it can be seen that the fragment concentration after a first rapid increase starts to decrease and then reaches an asymptotic value. In particular, after 120.0 min of reaction fragments, FR1, FR2, and FR3 reach a constant concentration of about 0.10, 0.19, and 0.32 mmol L^−1^, respectively.

The asymptotic character of the concentration profiles simulated for the hypothetical fragments is in line with the complete consumption of H_2_O_2_. The measured TOC concentration confirms the summarized TOC value, calculated from the changing organic carbon atom concentrations of the fragments. These fragments are fictitious; however, the stepwise degradation characterizes the dynamic balance of organic carbon atoms in line of the model hypotheses, correctly.

**Fig. 7:**
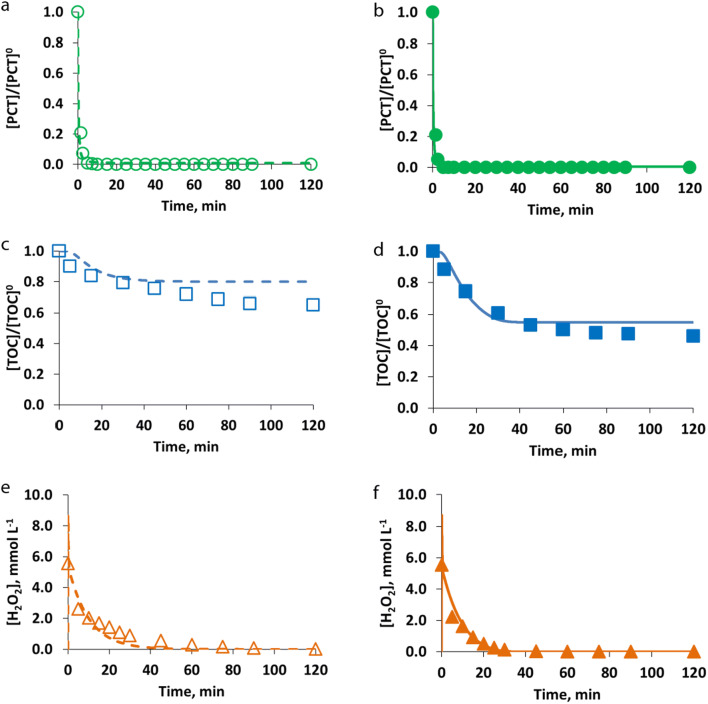
Model validation results: comparison of measured (symbols) and simulated (lines) values of PCT (circle, **a** and **b**) and TOC (square, **c** and **d**) presented as normalized values, and H_2_O_2_ (triangle, **e** and **f**) presented as concentration values (mmol L^−1^), obtained for [PCT]^0^ = 0.26 mmol L^−1^; [Fe^2+^]^0^ = 0.18 mmol L^−1^; [H_2_O_2_]^0^ = 5.56 mmol L^−1^ under dark (empty symbols and dashed lines, **a**, **c**, **e**) and irradiated (solid symbols and lines, **b**, **d**, **f**) conditions

Finally, an illustrative case of the model validation for the cases of EXP6_PCT and EXP15_PCT (see Table 2) is presented in Fig. 7. Specifically, the comparison of the simulated and measured data for the different model components (PCT, TOC, H_2_O_2_) obtained in the case of using [PCT]^0^ = 0.26 mmol L^−1^; [Fe^2+^]^0^ = 0.18 mmol L^−1^; [H_2_O_2_]^0^ = 5.56 mmol L^−1^ under dark and irradiated conditions, respectively, is reported.

Also in this case, RMSE was calculated as the average value of the RMSEs obtained for each experiment (dark and irradiated conditions) performed using PCT as the model compound. RMSE resulted in 30% for PCT, 7% for TOC, and 11% for H_2_O_2_, and showed the acceptable prediction of the model.

**Fig. 8:**
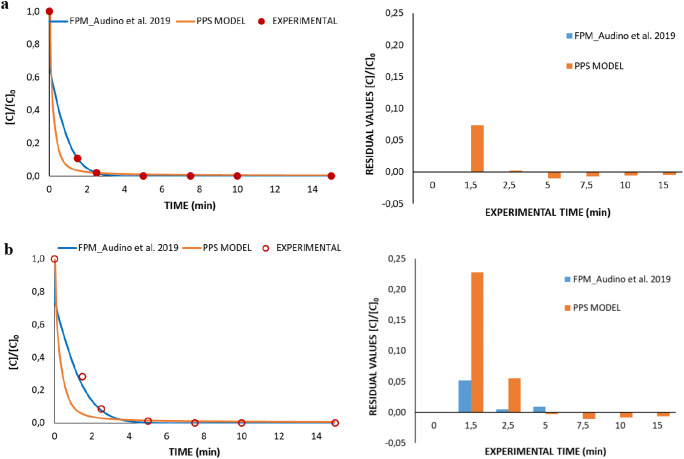
Comparative study illustrating the differences between the proposed model (PPS model, orange line) and a FPM proposed by Audino et al. 2019 (blue line), for two specific experiments, (**a**) the photo-Fenton experiment EXP18 (see Table 2) and the (**b**) Fenton experiment EXP8 (see Table 2), represented by solid and empty symbols respectively

In addition, a comparative study illustrating the differences between the proposed model and a FPM developed by Audino et al. (2019), for two specific experiments (the Fenton experiment EXP8 and the photo-Fenton experiment EXP18 in Table 2), is shown in Fig. 8a and b, respectively. The comparative study has been presented in a qualitative and quantitative way by means of the residual values. This shows the difference between experimental and predicted values by the proposed model (named PPS model in Fig. 8) and by FPM from Audino et al. (2019), represented in a bar diagram. It must be noticed that the model by Audino et al. was fitted to the same pilot plant described in Fig. 2 and using the same experimental results described in Table 2.

As can be observed, the proposed PPS model, compared with the more accurate FPM by Audino et al. (2019), slightly overestimates the PCT degradation both in the photo-Fenton (Fig. 8a) and in the Fenton (Fig. 8b) cases. However, the proposed PPS model produces differences below 1% in the photo-Fenton case and below 5% in the Fenton case, for a short time interval (2–5 min), and correctly describes the early removal of PCT. In addition, the proposed PPS model is also able to model TOC for the whole treatment span, which is not available in the FPM by Audino et al. (2019). The TOC modeling represents a very significant advantage of the proposed methodology since, as already underlined, it allows describing the overall quality of wastewater which can be composed of many targets.

### Estimation of the kinetic parameters of the degradation mechanism for the second target compound (SQX)

In this step, the experimental data derived by the assays performed using SQX as the model compound were used to validate the proposed methodology, previously fitted to the degradation of PCT.

Also, in this case, the kinetic parameters of the core mechanism, or rather *k*_0_, *k*_1_, *k*_2_, and *k*_elimination_, were not estimated and were assumed equal to those summarized in Table 6. Consequently, to validate the methodology using SQX, only the two kinetic parameters describing the first degradation step and the following ones needed to be estimated:

- ${k}_{\mathrm{target}}^{\mathrm{SQX}}$ [L(mmol s)^−1^] for the first degradation step of the target compound;
- ${k}_{\mathrm{fragments}}^{\mathrm{SQX}}$ [L(mmol s)^−1^] for the following degradation steps of the fragments.

Hence, as can be observed, the estimation strategy of the kinetic parameters describing the degradation mechanism of SQX is based on the same methodology followed for PCT. However, having the SQX a different number of carbon atoms and consequently a different number of breakage steps, compared with PCT, it was necessary to proceed to the estimation of the specific kinetic parameters of its degradation mechanism.

As for PCT, also in this case, parameter estimation strategy was tuned in order to give priority to a best fitting of TOC and H_2_O_2_ rather than the target and the specific SQX degradation mechanism. The estimated values of the relative kinetic parameters are summarized in Table 8.

**Table 8:** Degradation mechanism and estimated values of the related kinetics for SQX

Target compound: SQX (C_14_H_11_N_4_NaO_2_S)	
Degradation mechanism	
Formal fragmentation	14 → 7 → 3.5 → 1.75 → 0.875
TOC value	TOC = 14∙*c*_1_ + 7∙*c*_2_ + 3.5∙*c*_3_ + 1.75∙*c*_4_
Number of carbon atoms, NC	NC = 14
Number of the consecutive breakage steps, *B*	*B* = 5
Target-specific model parameters	
${k}_{\mathrm{target}}^{\mathrm{PCT}}$ = 4.5 × 102 L mol^−1^ s^−1^	
${k}_{\mathrm{fragments}}^{\mathrm{PCT}}$ = 3.5 × 102 L mol^−1^ s^−1^	

In this case, the rate of the first degradation step resulted to be similar to the rate of the subsequent degradation steps.

The degradability of SQX resulted to be obviously less than the one observed for PCT because of the greater carbon number, as well as a direct consequence of its structure.

**Fig. 9:**
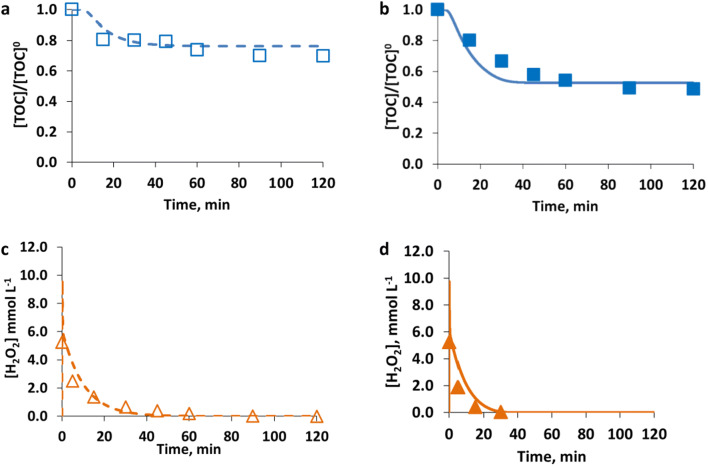
Model training results: comparison of measured (symbols) and simulated (lines) values of TOC (square, **a** and **b**) presented as normalized values, and H_2_O_2_ (triangle, **c** and **d**) presented as concentration values (mmol L^−1^), obtained for [SQX]^0^ = 0.08 mmol L^−1^; [Fe^2+^]^0^ = 0.18 mmol L^−1^; [H_2_O_2_]^0^ = 5.24 mmol L^−1^ under dark (empty symbols and dashed lines, **a** and **c**) and irradiated (solid symbols and lines, **b** and **d**) conditions

Only results of TOC and H_2_O_2_ are presented to discuss the SQX case because the main aim was to validate the proposed methodology as a general approach that can be applied for single or multiple targets in different wastewaters, whose quality can be described by a lumped parameter (as it is the TOC). Hence, Fig. 9 shows the comparison of the simulated and measured data of TOC and H_2_O_2_ obtained in the case of EXP2_SQX and EXP5_SQX (see Table 3). Specifically, these experiments refer to [SQX]^0^ = 0.08 mmol L^−1^; corresponding to [TOC]^0^ = 13.72 mg L^−1^, [Fe^2+^]^0^ = 0.18 mmol L^−1^; [H_2_O_2_]^0^ = 5.24 mmol L^−1^ under dark and irradiated conditions, respectively.

In this case, the enhancement of the process performance, promoted by the use of radiation, was more evident. In the specific case shown in Fig. 9, the SQX decayed below the detection limit of the equipment in about 60.0 min under irradiated conditions and in about 90.0 min under dark conditions (experimental results, not shown). Moreover, it can be seen that a 52% final TOC conversion was attained under irradiated conditions, conversely a 31% final TOC conversion was reached under dark conditions (experimental results).

Regarding H_2_O_2_, this was completely consumed under both dark and irradiated conditions, in about 40.0 and 30.0 min, respectively.

The RMSE averaged between all the RMSEs obtained for each experiment (dark and irradiated conditions) performed using SQX as the model compound, resulting in 17% for SQX, 26% for TOC, and 19% for H_2_O_2_, and showed that the model well approximates this system behavior.

The RMSE values slightly increased compared with the previous results obtained with PCT (30% for PCT, 7% for TOC, and 11% for H_2_O_2_). However, it must be taken into account that a smaller number of experiments (6 against 18) was employed.

It is important to notice that a minor number of experiments was necessary to validate the model (with SQX) than to train it (with PCT), since a smaller number of kinetic parameters needed to be estimated. This shows a clear advantage of the proposed simplified but generalizable methodology.

### Estimation of the kinetic parameters of the degradation mechanism for the third target compound (FA)

In this step, the experimental data derived by the assays performed with FA were used to validate the proposed methodology with a third model compound.

This model compound was selected in order to test the response of the model for the degradation of a very simple molecule.

As for PCT and SQX, also in this case, the kinetic parameters of the core mechanism, or rather *k*_0_, *k*_1_, *k*_2_, and *k*_elimination_, were not estimated and were assumed equal to those summarized in Table 6.

Moreover, even though the starting molecule of FA is composed of only one carbon atom, for coherence with the methodological approach defined for the first two targets, it was decided to take into account the attack to the parent compound and the attack to the intermediates originating from it.

Consequently, to validate the methodology using FA, the two kinetic parameters describing the first degradation step and the following ones needed to be estimated:

- ${k}_{\mathrm{target}}^{\mathrm{FA}}$[L(mmol s)^−1^] for the first degradation step of the target compound;
- ${k}_{\mathrm{intermediates}}^{\mathrm{FA}}$[L(mmol s)^−1^] for the following degradation steps of the intermediates.

In this case, it must be noticed that it was decided to only follow the TOC evolution due to the simplicity of the starting molecule of FA composed of only one carbon atom.

The specific FA degradation mechanism and the estimated values of the related kinetic parameters are summarized in the following Table 9.

**Table 9:** Degradation mechanism and estimated values of the related kinetics for FA

Target compound: FA (CH_2_O_2_)	
Degradation mechanism	
Formal fragmentation	1 → 1
TOC value	TOC = 1∙*c*_1_ + 1∙*c*_2_
Number of carbon atoms, NC	NC = 1
Number of the consecutive breakage steps, *B*	*B* = 1
Target-specific model parameters	
${k}_{\mathrm{target}}^{\mathrm{PCT}}$ = 65.0 × 10^-2^ L mol^−1^s^−1^	
${k}_{\mathrm{kintermediates}}^{\mathrm{FA}}$ = 7.5 × 10^-2^ L mol^−1^s^−1^	

As can be observed, the first step (attack to the parent compound) is characterized by a kinetic parameter of one order magnitude higher than the one describing the subsequent attack to the intermediates originating from the first degradation step. In this case, the parameter estimation suggests that the second degradation step could probably be avoided. However, this hypothesis needs to be experimentally confirmed by the evolution of formic acid during the treatment span (with HPLC analysis).

**Fig. 10:**
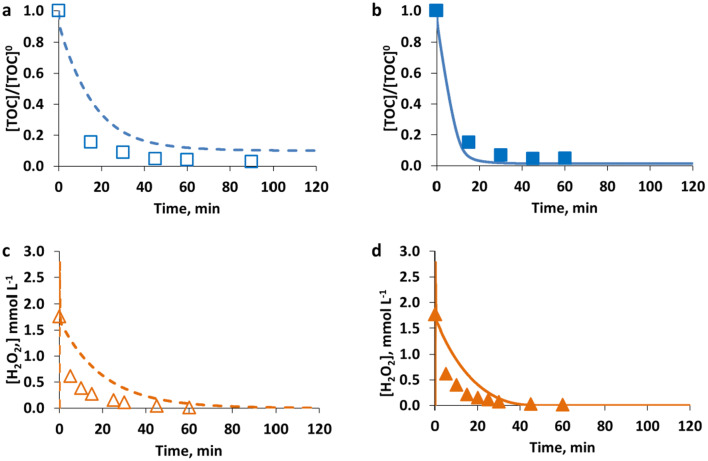
Model training results: comparison of measured (symbols) and simulated (lines) values of TOC (square, **a** and **b**) presented as normalized values, and H_2_O_2_ (triangle, **c** and **d**) presented as concentration values (mmol L^−1^), obtained for [FA]^0^ = 0.87 mmol L^−1^; [Fe^2+^]^0^ = 0.09 mmol L^−1^; [H_2_O_2_]^0^ = 1.76 mmol L^−1^ under dark (empty symbols and dashed lines, **a** and **c**) and irradiated (solid symbols and lines, **b** and **d**) conditions

Figure 10 shows the results of TOC and H_2_O_2_ and particularly the comparison between experimental and simulated data obtained in the case of EXP2_FA and EXP4_FA (see Table 4), particularly for [FA]^0^ = 0.87 mmol L^−1^; [Fe^2+^]^0^ = 0.09 mmol L^−1^; [H_2_O_2_]^0^ = 1.76 mmol L^−1^ under dark and irradiated conditions, respectively.

As can be observed in Fig. 10, due to the simplicity of the molecule of the parent compound, the Fenton and photo-Fenton degradation were both very fast. In both cases, experimental results show very similar results starting from the first analyzed sample (15.0 min) until the end of the treatment. After 15.0 min, 80% TOC conversion was attained under both dark and irradiated conditions while a final TOC conversion of about 96% was reached in both cases.

These experimental results suggest that, in order to capture the enhancement due to the use of radiation, more experimental measurements during the first minutes of the reaction are required. However, the TOC measurements can be performed only every 15.0 min (measuring time of the equipment). Hence, only detection via HPLC of the target molecule and of the produced intermediates can help describe the system behavior during the first minutes of the Fenton and photo-Fenton treatments of formic acid.

The model in this case overestimates the enhancement of the process performance due to the use of irradiation, describing the photo-Fenton process as the faster one. This result is probably due, as already observed, to the lack of experimental measurements during the first minutes of the reaction. Moreover, it must be noted that a very low number of experiments (five) was performed to validate the proposed methodology with this third compound.

Finally, also in this case, H_2_O_2_ is completely consumed under both dark and irradiated conditions in about 45.0 and 60.0 min, respectively.

The root main square error (RMSE) average value (averaged as in the previous cases between the RMSEs obtained for each experiment (dark and irradiated conditions) performed using FA as model compound) resulted in 16% for TOC and 18% for H_2_O_2_, and highlighted the acceptable prediction of the model.

As can be observed, after the third validation, the RMSE value for TOC decreased while the one related to H_2_O_2_ remained constant (if compared with the results obtained for the second model compound: 26% for TOC, 19% for H_2_O_2_), so showing an improvement of the estimation capability of the proposed methodology.

To conclude and summarize the results of the model fitting, Table 10 presents the RMSE values calculated for each of the three model components taken into account (target, TOC, oxidant) and for the three different targets (PCT, SQX, FA).

**Table 10:** RMSE values calculated for each of the three model components taken into account (target, TOC, oxidant) and for the three different targets (PCT, SQX, FA)

Root main square error (RMSE)				No. of experiments performed
	Target compound (%)	TOC (%)	H_2_O_2_ (%)	
Core mechanism	(Not measured)	(Not measured)	17	4
PCT	30	7	11	23
SQX	17	26	19	11
FA	(Not measured)	16	18	5

Despite the fewer number of experiments used to validate the proposed methodology with SQX rather than with PCT, it was possible to improve the prediction of the target compound. However, the RMSE associated with the predictions of TOC and H_2_O_2_ slightly increased.

**Fig. 11:**
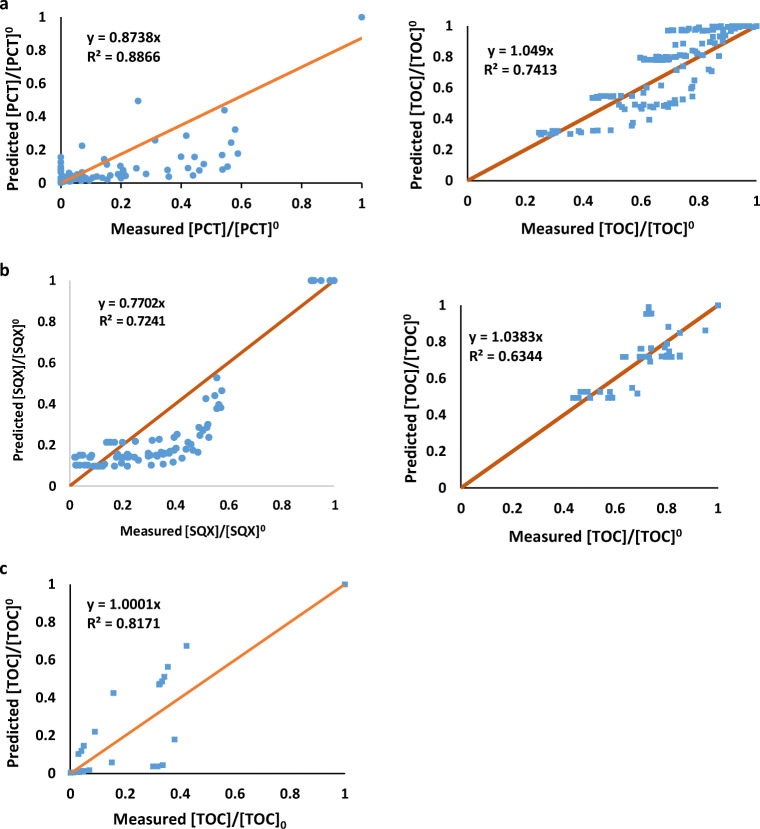
Predicted values versus the actual measured values, in the case of (**a**) the first selected target PCT and the relative TOC (**b**) the second selected target SQX and the relative TOC, and (**c**) the third selected target FA in which TOC and target is the same because of the single C atom

The acceptable fit of the model was finally illustrated by plotting the predicted values versus the actual measured values for each of the selected targets (PCT, SQX and FA) and the related TOC (see Fig. 11, a, b, and c).

As can be observed, Fig. 11 confirms the acceptable prediction of the model for both the specific selected targets and TOC.

## Conclusions

An innovative approach for modeling Fenton and photo-Fenton process remediated degradation of contaminants of emerging concern has been proposed and validated. The methodology allows developing models not only for the degradation of the target compound but also for the change of total organic carbon, which is used to define the overall quality of the wastewaters. The proposed model has reduced complexity and computational cost with the preservation of a fundamental comprehension of the underlying phenomena. Accordingly, the modeling approach offers a good generalization capability that enables addressing various compounds and wastewater systems.

The modeling methodology uses programmable process structures to develop and to implement a suitable process model. This methodology demonstrated the following advantages: (i) it supports fast model discovery by the automatized generation and configuration of various models from the description of the process network and from two general meta-prototypes; (ii) it supports the easy modification of the locally implementable declarative programs, associated with the state and transition prototypes; and (iii) the transition-based model representation helps the availability controlled normalized description of competitive fast processes.

The generalization capability of the methodology was successfully confirmed by the experimental validation of the developed model first for the degradation of paracetamol and next for the degradation of sulfaquinoxaline sodium salt and formic acid. Once the model is systematically created and validated to the first compound, the partial fitting of the model to further compounds was shown to require the adjustment of significantly fewer number of model parameters, which in turn needs less experimental data to produce the validation of the model at acceptable levels (RMSE).

The proposed model proved to be a causally interpretable and generalized candidate solution for the simulation-based analysis and design of Fenton and photo-Fenton reaction mediated degradation of various wastewaters that contain one or multiple target compounds.

Further research should include applying and validating the proposed methodology for multiple target compounds or mixtures of organic contaminants. Also, the simulation supported design of controlled multi-step or fed-batch dosage of H_2_O_2_ is to be studied to improve the decontamination ability of the process.
